# Ethyl (2*E*,4*Z*)-5-diethyl­amino-2-(phenyl­sulfon­yl)penta-2,4-dienoate

**DOI:** 10.1107/S1600536812023483

**Published:** 2012-05-31

**Authors:** Guo-Qiang Song, Pei-Pei Yu, Xian-Feng Huang

**Affiliations:** a School of Pharmaceutical Engineering and Life Science, Changzhou University, Changzhou 213164, People’s Republic of China

## Abstract

In the title compound, C_17_H_23_NO_4_S, the penta­diene group adopts a planar conformation, with an r.m.s. deviation of 0.0410 (14) Å. The phenyl ring makes a dihedral angle of 85.73 (11)° with the penta­diene group, while the penta­diene group makes dihedral angles of 11.38 (11) and 14.08 (10)°, respectively, with the amino and ester groups. In the crystal, molecules are linked *via* pairs of C—H⋯O inter­actions, forming inversion dimers.

## Related literature

For background information on penta­dienoates, see: Sorbetti *et al.* (2007 ▶). For structural data of penta­dienoates, see: Ceard *et al.* (2002 ▶). For details of weak hydrogen-bonding inter­actions, see: Steiner (2002 ▶).
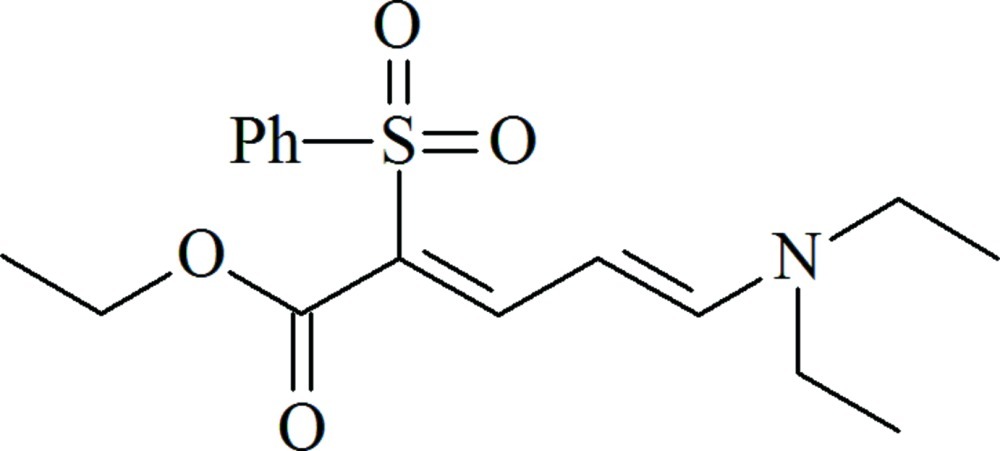


## Experimental

### 

#### Crystal data

C_17_H_23_NO_4_S
*M* *_r_* = 337.42
Monoclinic, 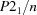

*a* = 14.489 (2) Å
*b* = 8.2989 (12) Å
*c* = 16.706 (3) Å
β = 114.313 (3)°
*V* = 1830.6 (5) Å^3^
*Z* = 4
Mo *K*α radiation
μ = 0.20 mm^−1^
*T* = 296 K
0.28 × 0.24 × 0.20 mm

#### Data collection

Bruker APEXII CCD area-detector diffractometer
Absorption correction: multi-scan (*SADABS*; Sheldrick, 2003 ▶) *T*_min_ = 0.669, *T*_max_ = 0.746
11618 measured reflections
4183 independent reflections
2977 reflections with *I* > 2σ(*I*)
*R*_int_ = 0.031

#### Refinement

*R*[*F* ^2^ > 2σ(*F* ^2^)] = 0.059
*wR*(*F* ^2^) = 0.168
*S* = 1.04
4183 reflections
211 parameters
H-atom parameters constrained
Δρ_max_ = 0.47 e Å^−3^
Δρ_min_ = −0.44 e Å^−3^

### 

Data collection: *APEX2* (Bruker, 2007 ▶); cell refinement: *APEX2* and *SAINT* (Bruker, 2007 ▶); data reduction: *SAINT*; program(s) used to solve structure: *SHELXTL* (Sheldrick, 2008 ▶); program(s) used to refine structure: *SHELXTL*; molecular graphics: *SHELXTL* and *DIAMOND* (Brandenburg, 2005 ▶); software used to prepare material for publication: *SHELXTL*.

## Supplementary Material

Crystal structure: contains datablock(s) global, I. DOI: 10.1107/S1600536812023483/pk2405sup1.cif

Structure factors: contains datablock(s) I. DOI: 10.1107/S1600536812023483/pk2405Isup2.hkl

Supplementary material file. DOI: 10.1107/S1600536812023483/pk2405Isup3.cml

Additional supplementary materials:  crystallographic information; 3D view; checkCIF report

## Figures and Tables

**Table 1:** Hydrogen-bond geometry (Å, °)

*D*—H⋯*A*	*D*—H	H⋯*A*	*D*⋯*A*	*D*—H⋯*A*
C13—H13*A*⋯O1^i^	0.93	2.41	3.277 (3)	155

Symmetry code: (i) 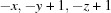.

**Fig. 1.**
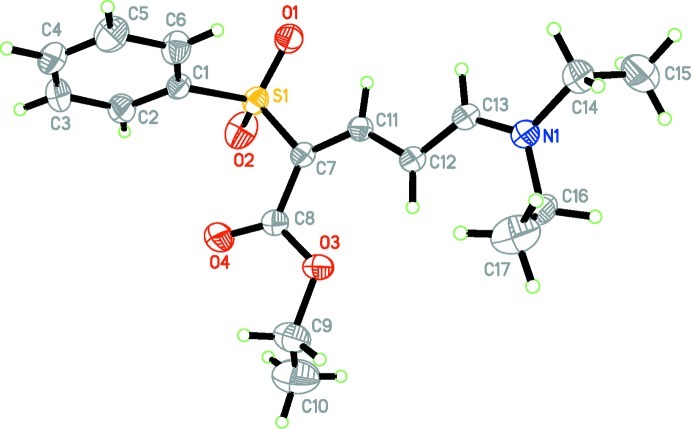
The molecular structure of (I) showing 30% probability displacement ellipsoids.

**Fig. 2.**
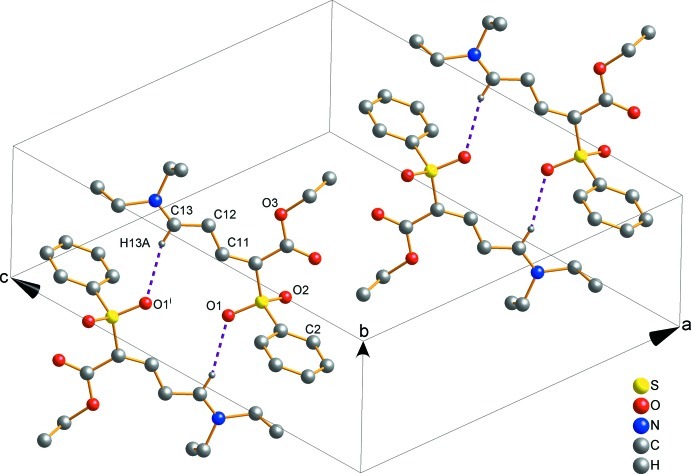
Crystal packing diagram of (I) with weak hydrogen bonds indicated as dashed lines.

## supplementary crystallographic information

### Comment

Analogues of pentadienoate have been used widely in perfumes, lubricants, as pharmaceutical intermediates and in other chemical industries (Ceard *et al.*, 2002; Sorbetti *et al.*, 2007). In this work, we report the crystal structure of the title compound, C_17_H_23_NO_4_S, (I). In the crystal (Fig. 1), the pentadiene group adopts a planar conformation with an r.m.s. deviation of 0.0410 (14) Å. The phenyl ring makes a dihedral angle of 85.73 (11)° with the pentadiene group. The dihedral angle of pentadiene with the amino group (atoms N1, C14, C16) is 11.38 (11)° and the dihedral angle between pentadiene and the ester group (atoms C8, O3, O4) is 14.08 (10)°. The crystal structure exhibits weak intermolecular C—H···O hydrogen bonds (Steiner, 2002; Table 1) between pairs of inversion related (-x, 1-y, 1-z) molecules to produce a weakly hydrogen-bonded dimer as shown in Fig. 2.

### Experimental

The title compound was prepared by stirring a mixture of malonaldehyde bis(dimethyl acetal) (411 mg, 2.5 mmol), ethanamine (366 mg, 2.5 mmol) and acetic acid (300 mg, 5 mmol) under reflux for 1 h. After cooling, ethyl 2-(phenylsulfonyl)acetate (428 mg, 1.87 mmol), DMF (1.5 ml) and 1,8-diazabicyclo[5.4.0]undec-7-ene (DBU, 761 mg, 5 mmol) were added and stirred for 6 h. Then the reaction mixture was added dropwise to ice water (20 ml) to give a yellow solid (259 mg), which was dissolved in 2-propanol. Pale yellow rhomboid-shaped crystals of (I) formed upon evaporation after 7d.

### Refinement

All the H atoms were positioned geometrically (C—H = 0.93–0.97 Å) and refined as riding, with *U*_iso_(H) = 1.2*U*_eq_(carrier) or 1.5*U*_eq_(methyl C).

### Crystal data

**Table:**

C_17_H_23_NO_4_S	*F*(000) = 720
*M**_r_* = 337.42	*D*_x_ = 1.224 Mg m^−^^3^
Monoclinic, *P*2_1_/*n*	Mo *K*α radiation, λ = 0.71073 Å
Hall symbol: -P 2yn	Cell parameters from 3554 reflections
*a* = 14.489 (2) Å	θ = 2.7–25.5°
*b* = 8.2989 (12) Å	µ = 0.20 mm^−^^1^
*c* = 16.706 (3) Å	*T* = 296 K
β = 114.313 (3)°	Block, pale yellow
*V* = 1830.6 (5) Å^3^	0.28 × 0.24 × 0.20 mm
*Z* = 4	

### Data collection

**Table:**

Bruker APEXII CCD area-detector diffractometer	4183 independent reflections
Radiation source: fine-focus sealed tube	2977 reflections with *I* > 2σ(*I*)
Graphite monochromator	*R*_int_ = 0.031
φ and ω scans	θ_max_ = 27.6°, θ_min_ = 1.6°
Absorption correction: multi-scan (*SADABS*; Sheldrick, 2003)	*h* = −18→18
*T*_min_ = 0.669, *T*_max_ = 0.746	*k* = −10→10
11618 measured reflections	*l* = −21→19

### Refinement

**Table:**

Refinement on *F*^2^	Primary atom site location: structure-invariant direct methods
Least-squares matrix: full	Secondary atom site location: difference Fourier map
*R*[*F*^2^ > 2σ(*F*^2^)] = 0.059	Hydrogen site location: inferred from neighbouring sites
*wR*(*F*^2^) = 0.168	H-atom parameters constrained
*S* = 1.04	*w* = 1/[σ^2^(*F*_o_^2^) + (0.0806*P*)^2^ + 0.7143*P*] where *P* = (*F*_o_^2^ + 2*F*_c_^2^)/3
4183 reflections	(Δ/σ)_max_ < 0.001
211 parameters	Δρ_max_ = 0.47 e Å^−^^3^
0 restraints	Δρ_min_ = −0.44 e Å^−^^3^

### Special details

**Table:**

Geometry. All e.s.d.'s (except the e.s.d. in the dihedral angle between two l.s. planes) are estimated using the full covariance matrix. The cell e.s.d.'s are taken into account individually in the estimation of e.s.d.'s in distances, angles and torsion angles; correlations between e.s.d.'s in cell parameters are only used when they are defined by crystal symmetry. An approximate (isotropic) treatment of cell e.s.d.'s is used for estimating e.s.d.'s involving l.s. planes.
Refinement. Refinement of *F*^2^ against ALL reflections. The weighted *R*-factor *wR* and goodness of fit *S* are based on *F*^2^, conventional *R*-factors *R* are based on *F*, with *F* set to zero for negative *F*^2^. The threshold expression of *F*^2^ > σ(*F*^2^) is used only for calculating *R*-factors(gt) *etc*. and is not relevant to the choice of reflections for refinement. *R*-factors based on *F*^2^ are statistically about twice as large as those based on *F*, and *R*- factors based on ALL data will be even larger.

### Fractional atomic coordinates and isotropic or equivalent isotropic displacement parameters (Å^2^)

**Table:**

	*x*	*y*	*z*	*U*_iso_*/*U*_eq_	
S1	0.11499 (4)	0.59041 (7)	0.39164 (4)	0.04777 (19)	
O1	0.02067 (12)	0.5770 (2)	0.40107 (12)	0.0639 (5)	
O2	0.12167 (17)	0.7075 (2)	0.33125 (13)	0.0767 (6)	
O3	0.38118 (13)	0.6802 (3)	0.58018 (13)	0.0790 (7)	
O4	0.33628 (15)	0.6001 (3)	0.44145 (14)	0.0809 (7)	
N1	0.22370 (15)	0.6412 (3)	0.79523 (13)	0.0586 (5)	
C1	0.13732 (16)	0.3988 (3)	0.35660 (15)	0.0445 (5)	
C2	0.1630 (2)	0.3849 (3)	0.28691 (19)	0.0635 (7)	
H2A	0.1709	0.4763	0.2581	0.076*	
C3	0.1772 (2)	0.2316 (4)	0.2595 (2)	0.0825 (10)	
H3A	0.1942	0.2202	0.2119	0.099*	
C4	0.1661 (2)	0.0986 (4)	0.3026 (2)	0.0787 (9)	
H4A	0.1764	−0.0032	0.2844	0.094*	
C5	0.1404 (3)	0.1130 (3)	0.3716 (2)	0.0789 (9)	
H5A	0.1338	0.0213	0.4009	0.095*	
C6	0.1240 (2)	0.2627 (3)	0.39856 (18)	0.0623 (7)	
H6A	0.1041	0.2724	0.4446	0.075*	
C7	0.21004 (16)	0.6228 (3)	0.49653 (15)	0.0455 (5)	
C8	0.31330 (19)	0.6342 (3)	0.50106 (17)	0.0572 (6)	
C9	0.4909 (2)	0.6711 (6)	0.5980 (3)	0.1000 (12)	
H9A	0.5311	0.6566	0.6604	0.120*	
H9B	0.5027	0.5796	0.5673	0.120*	
C10	0.5202 (4)	0.8158 (8)	0.5691 (4)	0.153 (2)	
H10A	0.5916	0.8128	0.5836	0.229*	
H10B	0.5053	0.9064	0.5975	0.229*	
H10C	0.4836	0.8258	0.5066	0.229*	
C11	0.18153 (17)	0.6212 (3)	0.56550 (15)	0.0453 (5)	
H11A	0.1130	0.6014	0.5493	0.054*	
C12	0.23716 (17)	0.6439 (3)	0.65536 (15)	0.0480 (5)	
H12A	0.3055	0.6710	0.6780	0.058*	
C13	0.18773 (18)	0.6250 (3)	0.70915 (16)	0.0522 (6)	
H13A	0.1197	0.5969	0.6813	0.063*	
C14	0.1598 (3)	0.6048 (4)	0.8424 (2)	0.0772 (9)	
H14A	0.1037	0.5372	0.8056	0.093*	
H14B	0.1993	0.5447	0.8953	0.093*	
C15	0.1196 (3)	0.7509 (5)	0.8664 (3)	0.1104 (14)	
H15A	0.0789	0.7212	0.8971	0.166*	
H15B	0.0788	0.8094	0.8141	0.166*	
H15C	0.1748	0.8175	0.9035	0.166*	
C16	0.32789 (19)	0.6914 (3)	0.84751 (16)	0.0595 (6)	
H16A	0.3300	0.7548	0.8970	0.071*	
H16B	0.3504	0.7597	0.8119	0.071*	
C17	0.3990 (3)	0.5521 (4)	0.8809 (3)	0.0984 (11)	
H17A	0.4649	0.5909	0.9193	0.148*	
H17B	0.4034	0.4962	0.8323	0.148*	
H17C	0.3744	0.4799	0.9126	0.148*	

### Atomic displacement parameters (Å^2^)

**Table:**

	*U*^11^	*U*^22^	*U*^33^	*U*^12^	*U*^13^	*U*^23^
S1	0.0517 (3)	0.0390 (3)	0.0470 (3)	0.0037 (2)	0.0147 (2)	−0.0007 (2)
O1	0.0429 (9)	0.0721 (12)	0.0661 (11)	0.0088 (8)	0.0118 (8)	−0.0138 (9)
O2	0.1097 (16)	0.0469 (10)	0.0628 (12)	0.0035 (10)	0.0246 (11)	0.0143 (9)
O3	0.0504 (10)	0.1203 (18)	0.0705 (12)	−0.0272 (11)	0.0292 (9)	−0.0313 (12)
O4	0.0694 (12)	0.1177 (18)	0.0694 (12)	−0.0237 (11)	0.0426 (10)	−0.0264 (12)
N1	0.0557 (12)	0.0719 (14)	0.0515 (12)	−0.0186 (10)	0.0256 (10)	−0.0151 (10)
C1	0.0456 (11)	0.0395 (11)	0.0466 (12)	−0.0035 (9)	0.0172 (9)	−0.0058 (9)
C2	0.0724 (17)	0.0638 (16)	0.0657 (16)	−0.0099 (13)	0.0399 (14)	−0.0077 (13)
C3	0.088 (2)	0.092 (2)	0.090 (2)	−0.0117 (18)	0.0583 (19)	−0.0339 (19)
C4	0.0771 (19)	0.0543 (16)	0.112 (3)	−0.0126 (14)	0.0456 (19)	−0.0323 (17)
C5	0.095 (2)	0.0443 (15)	0.104 (2)	−0.0059 (14)	0.047 (2)	−0.0028 (15)
C6	0.0828 (19)	0.0460 (13)	0.0643 (16)	−0.0011 (12)	0.0366 (14)	0.0006 (12)
C7	0.0447 (11)	0.0422 (11)	0.0499 (12)	−0.0043 (9)	0.0197 (10)	−0.0079 (9)
C8	0.0533 (13)	0.0644 (15)	0.0590 (15)	−0.0143 (12)	0.0284 (12)	−0.0131 (12)
C9	0.0607 (18)	0.154 (4)	0.091 (2)	−0.019 (2)	0.0365 (18)	−0.023 (3)
C10	0.114 (4)	0.185 (6)	0.178 (5)	−0.061 (4)	0.079 (4)	−0.030 (4)
C11	0.0413 (11)	0.0406 (11)	0.0544 (13)	−0.0010 (9)	0.0203 (10)	−0.0077 (10)
C12	0.0435 (11)	0.0493 (12)	0.0522 (13)	−0.0061 (10)	0.0205 (10)	−0.0104 (10)
C13	0.0470 (12)	0.0545 (14)	0.0549 (13)	−0.0104 (10)	0.0208 (10)	−0.0143 (11)
C14	0.082 (2)	0.092 (2)	0.0699 (18)	−0.0248 (17)	0.0428 (16)	−0.0180 (16)
C15	0.102 (3)	0.121 (3)	0.137 (3)	−0.019 (2)	0.079 (3)	−0.033 (3)
C16	0.0630 (15)	0.0623 (16)	0.0482 (13)	−0.0171 (12)	0.0177 (11)	−0.0118 (12)
C17	0.080 (2)	0.078 (2)	0.115 (3)	−0.0047 (18)	0.018 (2)	0.005 (2)

### Geometric parameters (Å, º)

**Table:**

S1—O2	1.4324 (19)	C9—C10	1.423 (7)
S1—O1	1.4429 (18)	C9—H9A	0.9700
S1—C7	1.747 (2)	C9—H9B	0.9700
S1—C1	1.770 (2)	C10—H10A	0.9600
O3—C8	1.338 (3)	C10—H10B	0.9600
O3—C9	1.494 (4)	C10—H10C	0.9600
O4—C8	1.206 (3)	C11—C12	1.394 (3)
N1—C13	1.319 (3)	C11—H11A	0.9300
N1—C16	1.459 (3)	C12—C13	1.369 (3)
N1—C14	1.473 (3)	C12—H12A	0.9300
C1—C2	1.365 (3)	C13—H13A	0.9300
C1—C6	1.384 (3)	C14—C15	1.471 (5)
C2—C3	1.396 (4)	C14—H14A	0.9700
C2—H2A	0.9300	C14—H14B	0.9700
C3—C4	1.363 (5)	C15—H15A	0.9600
C3—H3A	0.9300	C15—H15B	0.9600
C4—C5	1.355 (5)	C15—H15C	0.9600
C4—H4A	0.9300	C16—C17	1.495 (4)
C5—C6	1.375 (4)	C16—H16A	0.9700
C5—H5A	0.9300	C16—H16B	0.9700
C6—H6A	0.9300	C17—H17A	0.9600
C7—C11	1.375 (3)	C17—H17B	0.9600
C7—C8	1.470 (3)	C17—H17C	0.9600
			
O2—S1—O1	118.10 (12)	C9—C10—H10A	109.5
O2—S1—C7	110.37 (12)	C9—C10—H10B	109.5
O1—S1—C7	107.05 (11)	H10A—C10—H10B	109.5
O2—S1—C1	107.58 (11)	C9—C10—H10C	109.5
O1—S1—C1	106.07 (11)	H10A—C10—H10C	109.5
C7—S1—C1	107.10 (10)	H10B—C10—H10C	109.5
C8—O3—C9	118.1 (2)	C7—C11—C12	131.4 (2)
C13—N1—C16	121.9 (2)	C7—C11—H11A	114.3
C13—N1—C14	120.6 (2)	C12—C11—H11A	114.3
C16—N1—C14	117.5 (2)	C13—C12—C11	117.7 (2)
C2—C1—C6	120.5 (2)	C13—C12—H12A	121.2
C2—C1—S1	120.57 (19)	C11—C12—H12A	121.2
C6—C1—S1	118.88 (18)	N1—C13—C12	128.7 (2)
C1—C2—C3	119.0 (3)	N1—C13—H13A	115.7
C1—C2—H2A	120.5	C12—C13—H13A	115.7
C3—C2—H2A	120.5	C15—C14—N1	112.5 (3)
C4—C3—C2	120.0 (3)	C15—C14—H14A	109.1
C4—C3—H3A	120.0	N1—C14—H14A	109.1
C2—C3—H3A	120.0	C15—C14—H14B	109.1
C5—C4—C3	120.7 (3)	N1—C14—H14B	109.1
C5—C4—H4A	119.6	H14A—C14—H14B	107.8
C3—C4—H4A	119.6	C14—C15—H15A	109.5
C4—C5—C6	120.2 (3)	C14—C15—H15B	109.5
C4—C5—H5A	119.9	H15A—C15—H15B	109.5
C6—C5—H5A	119.9	C14—C15—H15C	109.5
C5—C6—C1	119.5 (3)	H15A—C15—H15C	109.5
C5—C6—H6A	120.3	H15B—C15—H15C	109.5
C1—C6—H6A	120.3	N1—C16—C17	112.8 (2)
C11—C7—C8	127.5 (2)	N1—C16—H16A	109.0
C11—C7—S1	117.03 (17)	C17—C16—H16A	109.0
C8—C7—S1	115.27 (17)	N1—C16—H16B	109.0
O4—C8—O3	122.8 (2)	C17—C16—H16B	109.0
O4—C8—C7	124.3 (2)	H16A—C16—H16B	107.8
O3—C8—C7	112.8 (2)	C16—C17—H17A	109.5
C10—C9—O3	109.3 (4)	C16—C17—H17B	109.5
C10—C9—H9A	109.8	H17A—C17—H17B	109.5
O3—C9—H9A	109.8	C16—C17—H17C	109.5
C10—C9—H9B	109.8	H17A—C17—H17C	109.5
O3—C9—H9B	109.8	H17B—C17—H17C	109.5
H9A—C9—H9B	108.3		
			
O2—S1—C1—C2	4.3 (2)	C1—S1—C7—C8	64.6 (2)
O1—S1—C1—C2	131.5 (2)	C9—O3—C8—O4	−7.0 (5)
C7—S1—C1—C2	−114.4 (2)	C9—O3—C8—C7	170.3 (3)
O2—S1—C1—C6	−172.9 (2)	C11—C7—C8—O4	163.0 (3)
O1—S1—C1—C6	−45.7 (2)	S1—C7—C8—O4	−11.1 (4)
C7—S1—C1—C6	68.4 (2)	C11—C7—C8—O3	−14.3 (4)
C6—C1—C2—C3	−1.0 (4)	S1—C7—C8—O3	171.59 (19)
S1—C1—C2—C3	−178.2 (2)	C8—O3—C9—C10	86.6 (4)
C1—C2—C3—C4	−0.5 (5)	C8—C7—C11—C12	6.7 (4)
C2—C3—C4—C5	0.7 (5)	S1—C7—C11—C12	−179.3 (2)
C3—C4—C5—C6	0.7 (5)	C7—C11—C12—C13	−176.0 (2)
C4—C5—C6—C1	−2.2 (5)	C16—N1—C13—C12	2.6 (4)
C2—C1—C6—C5	2.4 (4)	C14—N1—C13—C12	−175.8 (3)
S1—C1—C6—C5	179.6 (2)	C11—C12—C13—N1	−179.4 (2)
O2—S1—C7—C11	132.99 (19)	C13—N1—C14—C15	−101.9 (3)
O1—S1—C7—C11	3.2 (2)	C16—N1—C14—C15	79.6 (4)
C1—S1—C7—C11	−110.18 (18)	C13—N1—C16—C17	−91.9 (3)
O2—S1—C7—C8	−52.3 (2)	C14—N1—C16—C17	86.6 (3)
O1—S1—C7—C8	177.97 (18)		

### Hydrogen-bond geometry (Å, º)

**Table:**

*D*—H···*A*	*D*—H	H···*A*	*D*···*A*	*D*—H···*A*
C13—H13*A*···O1^i^	0.93	2.41	3.277 (3)	155

Symmetry code: (i) −*x*, −*y*+1, −*z*+1.
